# Unmasking Myopericarditis Behind an ST-Segment Elevation Myocardial Infarction (STEMI) Presentation

**DOI:** 10.7759/cureus.87226

**Published:** 2025-07-03

**Authors:** Lazaro Basart, Oscar Diaz, Jasmandeep Bhandal, Montadar Mohana, Kevin Sande, Mariano Razzeto

**Affiliations:** 1 Internal Medicine, Palmetto General Hospital, Hialeah, USA; 2 Dr. Kiran C. Patel College of Osteopathic Medicine, Nova Southeastern University, Fort Lauderdale, USA

**Keywords:** cardiac cath, internal medicine-cardiology, myopericarditis, right bundle branch block, st-elevation myocardial infarction (stemi)

## Abstract

Myopericarditis is an inflammatory cardiac condition that can closely mimic ST-elevation myocardial infarction (STEMI), presenting with chest pain, elevated troponin levels, and ST-segment changes on electrocardiogram (ECG). We present the case of a 46-year-old man with a history of hypertension who presented to the emergency department with sudden-onset, substernal chest pain that awoke him from sleep. The pain was described as crushing in nature, associated with diaphoresis, and was initially attributed to anxiety. Electrocardiography revealed ST-segment elevations in leads II, III, and aVF, with reciprocal changes and an incomplete right bundle branch block (IRBBB). Initial troponin I was markedly elevated at 16.9 ng/mL. Given these concerning findings, the patient underwent emergent cardiac catheterization. Coronary angiography revealed no obstructive coronary artery disease, and left ventriculography demonstrated preserved systolic function. Further evaluation uncovered a recent viral upper respiratory infection, and transthoracic echocardiography showed diastolic dysfunction with a trivial pericardial effusion. The combination of clinical presentation, elevated cardiac markers, ST-segment changes, and absence of coronary pathology led to the diagnosis of myopericarditis. This case highlights the importance of maintaining a broad differential diagnosis in patients presenting with apparent acute coronary syndromes and underscores the need for comprehensive assessment to avoid unnecessary interventions.

## Introduction

Chest pain accounts for approximately 5% of visits to emergency departments in the USA [1]. Given its broad differential diagnosis, rapid identification of life-threatening causes is essential to guide emergent management. Among these, acute coronary syndrome (ACS), particularly ST-elevation myocardial infarction (STEMI), is a primary concern due to its high morbidity and mortality.

However, while ST-segment elevation on electrocardiogram (ECG) is a sensitive indicator of myocardial injury, it lacks specificity and may be seen in various cardiac and non-cardiac conditions [2]. As such, it is therefore crucial for clinicians to recognize mimics of acute MI, especially when the presentation does not entirely conform to classic ischemic patterns.

Acute pericarditis, defined as inflammation of the pericardium, is a notable cause of non-ischemic chest pain with ST-segment changes, often making it difficult to distinguish from acute MI [3]. When inflammation extends to involve both the myocardium and pericardium, the condition is termed myopericarditis. This diagnosis is particularly relevant in the setting of recent viral infections, where inflammatory cardiac conditions may closely resemble STEMI in both symptoms and electrocardiographic findings. In the case presented, the final diagnosis of myopericarditis was made based on clinical presentation, laboratory data, and imaging results.

## Case presentation

A 46-year-old man with a past medical history of hypertension presented to the emergency department (ED) with sudden-onset substernal chest pain that began 24 hours earlier while he was sleeping. The pain was described as crushing in nature, radiating to the left arm, and accompanied by diaphoresis. Initially, the patient attributed his symptoms to anxiety triggered by recent emotional stressors. However, as the pain persisted, he sought medical attention. He also reported a sore throat and a resolving cough two days prior to presentation. He denied tobacco or illicit drug use, history of deep vein thrombosis or pulmonary embolism, and had no prior episodes of chest pain.

**Figure 1:**
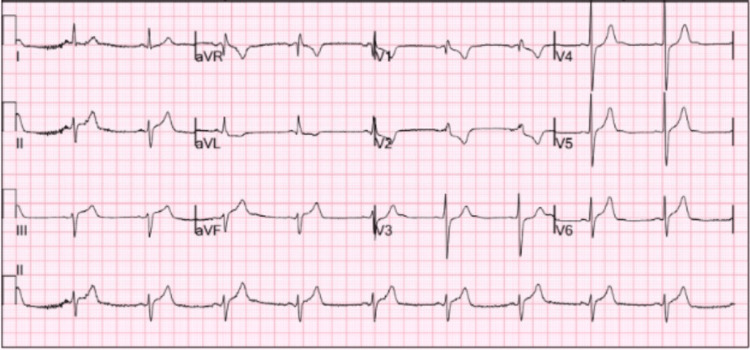
Electrocardiogram showing sinus rhythm with ST-segment elevations in leads II, III, and aVF, along with reciprocal ST-segment depressions in leads I and aVL. An incomplete right bundle branch block pattern is also noted in leads V1–V2. These findings are consistent with an inferior STEMI pattern.

On arrival, the patient appeared diaphoretic and in visible distress due to chest discomfort. Vital signs revealed sinus tachycardia with a heart rate of 133 beats per minute. ECG showed ST-segment elevations in leads II, III, and aVF, with reciprocal depressions in leads I and aVL, as well as an incomplete right bundle branch block (IRBBB) in leads V1-V2, findings suggestive of an inferior STEMI, as shown in Figure 1. Although IRBBB was noted, it is a nonspecific finding and did not play a key role in differentiating STEMI from myopericarditis in this case. Initial serum troponin I level was markedly elevated at 16.9 ng/mL (reference range: 0.012-0.034 ng/mL), as shown in Table 1. Given the concerning ECG findings, elevated cardiac biomarkers, and ongoing chest pain, the case was promptly discussed with the ED attending and interventional cardiologist. The patient was taken emergently for cardiac catheterization.

**Table 1:** Abnormal lab values on admission. The most significant abnormality was an elevated troponin I level of 16.9 ng/mL.

Abnormal lab values	Patient's lab values	Reference value
Aspartate transaminase (AST)	128 U/L	17-59 U/L
Troponin I	16.900 ng/mL	0.012-0.034 ng/mL
N-Terminal pro-B-type natriuretic peptide	1010 pg/mL	0-299 pg/mL
D-Dimer	0.323 ng/mL	0-499 ng/mL
Potassium	5.3 mmol/L	3.4-5.0 mmol/L
Glucose	125 mg/dL	74.0-106.0 mg/dL
Carbon dioxide	18 mmol/L	22-30 mmol/L

Additional laboratory workup included a D-dimer test, which was within normal limits, reducing the likelihood of pulmonary embolism as a cause of the patient’s tachycardia and chest pain. Although inflammatory markers such as CRP or ESR were not explicitly mentioned, the diagnosis was supported by clinical features and imaging criteria consistent with myopericarditis.

**Figure 2:**
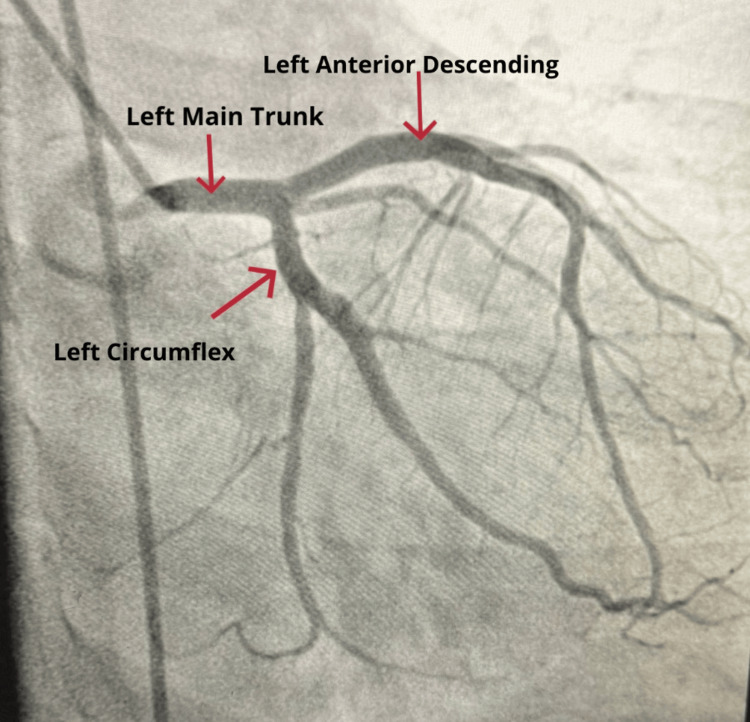
Cardiac catheterization showing no obstructive disease (NOD).

Coronary angiography revealed no obstructive coronary artery disease, as illustrated in Figure 2. Vascular access via the right femoral artery was uneventful, and closure was achieved using a vascular closure device. A transthoracic echocardiogram (TTE) performed later revealed a normal left ventricular size with mild concentric left ventricular hypertrophy. Diastolic function was consistent with Grade I (impaired relaxation) diastolic dysfunction, evidenced by a reversed E/A ratio. A small, trivial pericardial effusion was also noted. The estimated ejection fraction was 60%-65%. Given the clarity of the clinical picture and supportive echocardiographic findings, advanced imaging with cardiac magnetic resonance imaging (CMR) was not deemed necessary. While CMR is a valuable modality for confirming myocarditis, especially through late gadolinium enhancement and tissue characterization, its utility is most critical in cases of diagnostic uncertainty. In this case, the diagnosis of myopericarditis was deemed clinically sound without the need for further imaging.

Given the absence of obstructive coronary artery disease and the presence of viral prodromal symptoms, an inflammatory etiology was considered. The patient reported a recent upper respiratory infection with a severe cough a few days prior and noted only a sore throat on the day of presentation. Rapid influenza A/B and streptococcal antigen tests were negative. Further viral panel testing was not performed, as specific viral identification is not required for the diagnosis of myocarditis or myopericarditis and is generally not pursued due to its limited clinical utility. The patient was ultimately diagnosed with myopericarditis, which guided the treatment strategy. He was managed conservatively with nonsteroidal anti-inflammatory drugs (NSAIDs) and colchicine, in accordance with current clinical guidelines for the treatment of myopericarditis.

## Discussion

This case highlights the clinical challenge of differentiating between acute MI and myopericarditis, particularly in patients presenting with chest pain, elevated cardiac enzymes, and ST-segment changes. In this patient, the initial presentation strongly suggested MI; however, the absence of coronary artery obstruction on cardiac catheterization and the history of a recent viral illness raised clinical suspicion for myopericarditis. Viral myocarditis remains one of the major causes of acute and chronic (dilated) cardiomyopathy. Common viral etiologies include enteroviruses, particularly coxsackievirus B, as well as adenoviruses, parvoviruses, and certain herpesviruses [4]. The inflammatory pathophysiology of viral myocarditis may lead to significant myocardial injury and necrosis, even in the absence of ischemia.

In addition, influenza-associated pericarditis is a recognized but often underdiagnosed complication of influenza infections, as described in several case reports [5]. These cases can be misattributed to primary ischemic events if not evaluated thoroughly. Furthermore, a growing body of literature has described various cardiac manifestations, including myocarditis, pericarditis, acute coronary syndrome, and arrhythmias, following COVID-19 vaccination [6]. These associations underscore the emerging understanding of immune-mediated mechanisms involved in acute cardiac syndromes. 

Myopericarditis is distinguished from isolated pericarditis by evidence of myocardial involvement, which includes elevated cardiac biomarkers such as troponin and sometimes wall motion abnormalities. It differs from classic myocarditis in that it also involves pericardial inflammation, often evidenced by ST-segment elevations and occasionally small effusions. This overlap can make the diagnosis more challenging, especially when the presentation mimics acute coronary syndromes.

In this case, the diagnosis was further supported by echocardiographic findings of a small pericardial effusion and preserved systolic function, despite ECG findings that were nearly indistinguishable from those of a STEMI. Although the patient’s troponin I was markedly elevated at 16.9 ng/mL, higher than typically expected in viral myopericarditis, this level of elevation has been described in severe cases. Importantly, there were no wall motion abnormalities on echocardiography, and coronary angiography revealed no obstructive disease, strengthening the argument for an inflammatory rather than ischemic etiology. Although CMR is considered the gold standard for tissue characterization and can confirm the presence of myocardial inflammation, it was not pursued in this case. Our facility is a community hospital serving an underserved population, where advanced imaging such as CMR, though technically available, is reserved for cases where the diagnosis remains unclear. In this case, the combination of clinical presentation, markedly elevated biomarkers, absence of coronary obstruction, echocardiographic findings, and recent viral symptoms provided sufficient evidence to establish the diagnosis of myopericarditis. Furthermore, serial TTEs can be used to monitor cardiac function and pericardial effusion progression when CMR is not performed, offering a practical, non-invasive follow-up option in resource-limited settings. These findings stress the need for a broad differential diagnosis when evaluating chest pain in emergency settings, especially in patients without traditional cardiovascular risk factors.

## Conclusions

Myopericarditis is an important and sometimes underrecognized cause of chest pain that may closely mimic acute coronary syndromes, both clinically and electrocardiographically. This case underscores the diagnostic challenge of distinguishing myopericarditis from STEMI, especially in the presence of ST-segment elevations and significantly elevated troponin levels. The initial presentation of this 46-year-old man, with crushing chest pain, an elevated troponin level of 16.9 ng/mL, and ECG findings suggestive of inferior STEMI, highlighted this overlap.

Despite initial concerns for an acute ischemic event, the absence of obstructive coronary disease on angiography, combined with echocardiographic findings and a recent history of upper respiratory infection, pointed toward a diagnosis of probable viral myopericarditis. This case reinforces the importance of integrating clinical context, imaging, and invasive diagnostics to avoid misdiagnosis and unnecessary interventions. Clinicians should maintain a broad differential diagnosis, particularly in younger patients or those without traditional cardiovascular risk factors. Prompt recognition and appropriate management of myopericarditis are crucial for optimizing outcomes and preventing complications associated with inappropriate treatment.
